# Obstructive sleep apnoea: a cause of chronic cough

**DOI:** 10.1186/1745-9974-3-7

**Published:** 2007-07-02

**Authors:** Surinder S Birring, Alvin J Ing, Kevin Chan, Gavina Cossa, Sergio Matos, Michael DL Morgan, Ian D Pavord

**Affiliations:** 1 Respiratory Investigation Unit, Concord Hospital, Sydney, Australia; 2 Institute for Lung Health, Department of Respiratory Medicine, Glenfield Hospital, Leicester, UK; 3 Department of Respiratory Medicine, King's College Hospital, London, UK

## Abstract

Chronic cough is a common reason for presentation to both general practice and respiratory clinics. In up to 25% of cases, the cause remains unclear after extensive investigations. We report 4 patients presenting with an isolated chronic cough who were subsequently found to have obstructive sleep apnoea. The cough improved rapidly with nocturnal continuous positive airway pressure therapy. Further studies are required to investigate the prevalence of coexistence of these common conditions.

## Background

Chronic cough is one of the commonest reasons for presentation to respiratory clinics. Investigations are usually aimed at identifying the three most common causes of chronic cough: cough variant asthma, gastro-oesophageal reflux and upper airway cough syndrome. [1] In up to 25% of patients, the cause of cough remains unexplained after extensive investigations and treatment trials. [2-4] Patients experience considerable physical and psychological morbidity. Here, we report 4 well-characterised patients referred to a general respiratory clinic with unexplained chronic cough who were subsequently found to have obstructive sleep apnoea.

## Case presentations

### Patient 1

A 52-year-old financial advisor was referred by his general practitioner with a 3-month history of productive cough. He described a severe barking cough that occurred both in the day and night time and was exacerbated by lying flat, strong odours and smoky atmospheres. He was prescribed multiple courses of antibiotics, which were unhelpful. He also complained of mild dyspnoea on climbing hills but no wheezing. He had longstanding left nasal congestion without post-nasal drip following a broken nose in childhood and mild symptoms of gastro-oesophageal reflux. He had been diagnosed with hypertension and hypercholesterolaemia two years previously for which he was prescribed Bendroflumethiazide, Valsartan, Doxazoscin and Cervistatin. He was an ex-smoker and accumulated a 15 pack-year smoking history. His clinical examination and physical findings were normal. Initial spirometry and chest radiograph were within normal limits (Table 1). His cough was thought to be due to gastro-oesophageal reflux and rhinitis so he was started on a prolonged course of topical nasal steroids and high dose proton pump inhibitor. Methacholine airway challenge test, induced sputum eosinophil cell count, high resolution computerised tomography (HRCT) scan and echocardiogram arranged to investigate asthma, eosinophilic bronchitis, bronchiectasis and left ventricular dysfunction were all normal (Table 1).

**Table 1:** Patient characteristics

	**Patient 1**	**Patient 2**	**Patient 3**	**Patient 4**
**Age (years)**	52	73	46	63
**Gender (m/f)**	M	F	F	M
**Snoring**	Yes	Yes	Yes	Yes
**Nocturnal cough**	Yes	Yes	Yes	Yes
**Body mass Index (Kg/m^**2**^)**	35	32	37	33
**Baseline cough VAS**	90	95	50	80
**Post CPAP cough VAS**	0	5	0	0
**Cough duration at OSA diagnosis (mo)**	48	6	16	8
**FEV_**1 **_(%predicted)**	93	61	119	100
**FEV_**1**_/FVC (%)**	78	70	84	83
**PC_**20 **_mg/ml)**	>16	nd	>16	>16
**Sputum neutrophil (nr <65%)**	89	-	91	87
**Sputum eosinophil (nr <2%)**	0.3	-	1.4	0.5
**ESS preCPAP**	7	14	15	15
**ESS post CPAP**	3	8	7	5
**AHI baseline (per hour)**	36	86	62	10 (4% SpO_2 _dips)
**SpO_**2 **_(4% dips/hour) on CPAP**	0.7	0	3	0
**CPAP (cm H_**2**_o)**	12	12	8	6
**CPAP compliance (mean hours/night)**	6	4	7	6

NR: normal range; VAS: visual analogue score (0–100 mm worst); CPAP: continuous positive airway pressure; OSA: obstructive sleep apnoea; FEV_1_: forced expiratory volume; FVC: forced vital capacity; PC_20_: provocative concentration of methacholine causing >20% decline in FEV_1_; ESS: daytime Epworth Sleepiness Scale (range 0–24, nr<11); AHI: apnoea hypopnoea index (nr< 5/hour); SpO_2_: oxygen saturation

On review 6 and 12 months later his symptoms of rhinitis and gastro-oesophageal reflux had resolved but his cough persisted. No explanation for the cough was found and he discharged back to his general practitioner. He was re-referred back to the clinic 3 years later with daytime somnolence, lethargy, apnoeas and cough. In retrospect, he had mild daytime somnolence and lethargy at the time of initial presentation. He was noted to have a small oropharynx on external examination. Polysomnography was arranged that was consistent with the diagnosis of obstructive sleep apnoea (Table 1). Nocturnal nasal continuous positive airway pressure (CPAP) therapy was commenced and he noticed an immediate improvement in cough and daytime somnolence. His cough resolved entirely within 6 weeks and he remains free of cough 12 months later. The initiation of CPAP therapy has also led to a reduction in anti-hypertensive medications and complete resolution of oxygen desaturation on repeat sleep study.

### Patient 2

**Figure 1:**
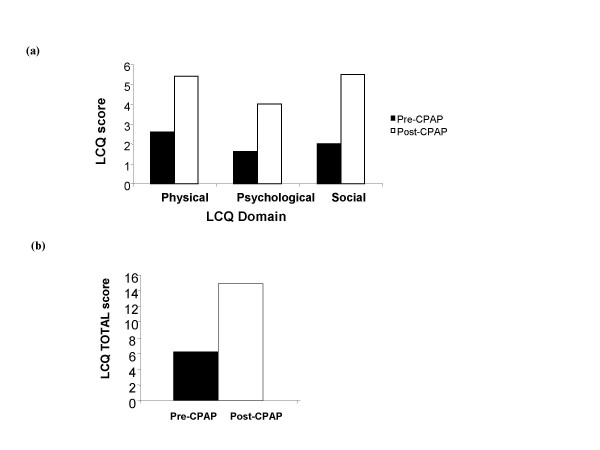
Improvement in cough specific quality of life after (continuous positive airway pressure) CPAP therapy in patient 2. (a) Leicester Cough Questionnaire (LCQ) Domain scores. (Higher score = better quality of life: QOL) (b) LCQ total score. (Minimal important difference 1.3; higher score = better QOL).

**Figure 2:**
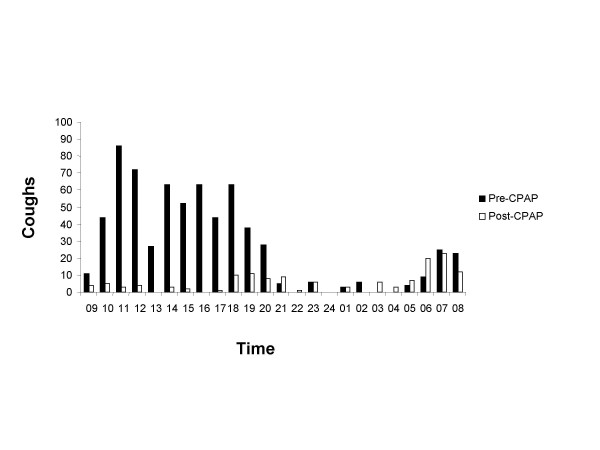
Decrease in cough frequency following CPAP in patient 2 assessed with the Leicester Cough Monitor (LCM).

A 73 year-old housewife with an 18-month history of severe chronic cough was referred by her respiratory physician for a second opinion. The cough was predominantly dry, interfered with her daily activities and occasionally disturbed her sleep. She did not report other respiratory symptoms, postnasal drip or gastro-oesophageal reflux. Her past medical history consisted of diabetes, hypertension, atrial fibrillation and aorto-femoral bypass for peripheral vascular disease. Soon after the onset of cough, she was diagnosed as having severe obstructive sleep apnoea with full polysomnography after admitting to symptoms of daytime somnolence, snoring and disturbed sleep (Table 1). She could not be persuaded to try CPAP therapy or alternative options and was more concerned about the chronic cough. She was an ex-smoker and stopped smoking 13-years previously, accumulating a 40-pack year smoking history. Clinical examination and chest radiograph were normal. Spirometry was consistent with a moderate restrictive defect consistent with obesity. Treatment trials with inhaled corticosteroids, short and long acting bronchodilators and high dose proton pump inhibitors were unsuccessful. Nasendoscopy, Ear-Nose-Throat evaluation, echocardiogram (normal left ventricular function) and bronchoscopy were normal. She had marked impairment of quality of life secondary to chronic cough as assessed with the Leicester Cough Questionnaire (Figure 1) and increased cough frequency measured with the Leicester Cough Monitor, a 24-hour ambulatory automated cough detection monitor (Figure 2). [5-7] She agreed to try CPAP therapy at a subsequent follow-up clinic. She was admitted for a CPAP titration study and a pressure of 12 cm H_2_o abolished snoring and apnoeas. She noticed a significant improvement in cough at the end of the first week on CPAP therapy. There was an 80% reduction in objective cough frequency at 6-weeks (672 vs 141 coughs per 24-hours) and a significant improvement in cough specific quality of life (Improvement in LCQ total score 8.2 which was greater than the LCQ minimal important difference of 1.3; Figures 1 and 2). She was able to resume regular activities after improvement of her cough.

### Patient 3

A 46-year-old nurse was referred by her occupational health physician with a 1-year history of productive cough. The cough occurred through the day and night and there were no specific triggers or associated symptoms. There were no symptoms of dyspnoea or wheeze. She did not complain of symptoms suggestive of rhinitis or gastro-oesophageal reflux. A trial of inhaled corticosteroids and nasal steroids were unhelpful. Her husband commented that she recently started snoring but he had not witnessed apnoeas. She complained of lethargy that was associated with mild somnolence. She had pneumonia in childhood but was unable to give further details. Her past medical history was otherwise unremarkable. She had never smoked and did not consume alcohol. On examination, she had evidence of retrognathia but was otherwise unremarkable. Chest radiograph and spirometry were within normal limits.

The initial differential diagnosis was cough secondary to asthma, eosinophilic bronchitis or bronchiectasis. Methacholine airway challenge test, induced sputum eosinophil cell count and HRCT were arranged and were normal (Table 1). Partial polysomnography (RM60 study: airflow, chest wall movement, oximetry, snoring, pulse) was subsequently arranged because of mild somnolence and snoring, which was highly suggestive of obstructive sleep apnoea. Nocturnal nasal CPAP therapy was commenced and she noticed an immediate improvement in cough, lethargy and somnolence. The cough resolved within 5 days and she remains free of cough 15 months later. Repeat sleep study indicates complete resolution of oxygen desaturation on CPAP.

### Patient 4

A 63-year-old office worker with the police force was referred by his general practitioner with a 4-month history of chronic productive cough. The cough was severe, occurred both day and night time and was worse lying flat. There were no dyspnoea, wheeze or symptoms suggesting gastro-oesophageal reflux and rhinitis. He was an ex-smoker with less than 2 pack year smoking history and did not consume alcohol. His past medical history was unremarkable. Clinical examination, chest radiograph and spirometry were normal.

The cause of cough was not clear. Methacholine airway challenge test and induced sputum eosinophil cell count were normal (Table 1). At 4-month review he complained of snoring and daytime somnolence. A pulse oximetry sleep study was arranged which was consistent with a diagnosis of obstructive sleep apnoea. The cough and sleepiness improved significantly with nocturnal nasal CPAP after 2 days and the cough resolved completely 3 months later. At 15-month review after initiation of CPAP therapy, the patient does not complain of cough or somnolence.

## Discussion

We report for the first time, four adult patients with obstructive sleep apnoea that presented with a chronic cough. All patients had a rapid improvement of cough with CPAP therapy and are free of respiratory symptoms twelve months later. This suggests a link between cough and obstructive sleep apnoea in these patients.

Obstructive sleep apnoea was not apparent at presentation in our patients. The initial investigations were directed at determining the aetiology of chronic cough using a standardised diagnostic algorithm. [8] These patients underwent assessment for the most common causes of chronic cough in non-smokers with normal chest radiograph and spirometry which are considered to be asthma, rhinitis and gastro-oesophageal reflux disease. None of our patients were taking angiotensin converting enzyme inhibitors. Daytime somnolence was present at initial consultation but this was not reported by patients or recognised by the physician. This symptom may have been masked by the severity of the cough and not considered by the physician since obstructive sleep apnoea is not a recognised cause of chronic cough. It is only when typical symptoms of obstructive sleep apnoea became apparent or progressed that polysomnography was requested and the diagnosis of obstructive sleep apnoea established. The lack of clinical suspicion of obstructive sleep apnoea at presentation with cough led to considerable delays in diagnosis and was over 3 years in one patient. Once CPAP therapy was initiated, there was a rapid improvement of cough and symptoms of obstructive sleep apnoea within days. This is consistent with a case report of a three-year-old boy with chronic cough, snoring and upper airway obstruction on polysomnography in whom there was resolution of cough after commencing CPAP therapy. [9]

Patients with obstructive sleep apnoea and cough are likely to have upper airway injury and inflammation resulting from snoring and frequent episodes of airway obstruction. Snoring and obstructive sleep apnoea cause airway epithelial damage and inflammatory cell infiltration of the lamina propia. [10] Our patients had a raised sputum neutrophil count consistent with inflammation in the large airways. [11] Patients with obstructive sleep apnoea have raised concentrations of inflammatory mediators in the upper airways that may sensitize cough receptors leading to heightened cough reflex sensitivity analogous to that seen in cough due to asthma and eosinophilic bronchitis. [12,13] Interestingly, subjects who snore are more likely to report a cough supporting the mechanism of airway injury causing cough in obstructive sleep apnoea. [14-18] It is possible that the cough may have resulted from mechanical causes and independently of airway inflammation since the effect of CPAP was rapid. Bonnet et al have described 5 patients with nocturnal cough and increased airway collapsibility secondary to bronchomalacia that responded to CPAP therapy. [19] It is possible that this condition may have co-existed in our patients with obstructive sleep apnoea.

Another potentially important mechanism of cough in patients with obstructive sleep apnoea is gastro-oesophageal reflux associated cough. Obstructive apnoea episodes increase trans-diaphragmatic pressure that may lead to insufficiency of the gastric cardia and lower oesophageal sphincter. [20] There is a higher incidence of gastro-oesophageal reflux in patient with obstructive sleep apnoea and CPAP therapy has been shown to reduce episodes of gastro-oesophageal reflux. [21,22] Another possibility is that CPAP therapy may have be effective at reducing nocturnal gastro-oesophageal reflux and associated cough independent of the presence of obstructive sleep apnoea. Only patient 1 reported symptoms of gastro-oesophageal reflux but his cough persisted despite a trial of high dose proton pump inhibitor. Gastro-oesophageal reflux associated cough cannot be categorically excluded in our patients because we did not measure 24-hour oesophageal pH or assess for the presence of non-acid gastro-oesophageal reflux and this requires further study. Another possible cause of cough in our patients is rhinitis although none had symptoms or evidence of rhinitis on external examination at the time of diagnosis. However, "silent" rhinitis cannot be fully excluded in our patients. Finally, a general abnormality of upper airway reflexes is possible that leads to reduced upper airway tone and calibre and loss of inhibitory pathways of the cough reflex. [23]

Limitations of our study are the small number of patients studied and the diagnoses of obstructive sleep apnoea were based on limited polysomnography and oximetry study in two cases. It is unlikely that these studies are false positives since both patients did not have a history of chronic obstructive pulmonary disease or heart failure where false positive studies are often seen. Furthermore, these studies are considered acceptable first line diagnostic studies in recent guidelines. [24] We are confident that our patients had obstructive sleep apnoea since they had suggestive symptoms and good clinical and objective response to CPAP therapy. We were able to utilise recently available objective cough assessment tools in patient 2 to validate the presence of a frequent cough associated with impaired quality of life and a clinically significant improvement with CPAP therapy. [5-7] This study suggests that objective coughing monitoring tools may be useful and responsive in patients with chronic cough and this requires confirmation in larger numbers.

Cough is likely to be a common symptom in patients presenting with obstructive sleep apnoea. The prevalence of obstructive sleep apnoea in patients presenting with a chronic cough is not known and deserves further study. The cause of cough remains unexplained in up to 30% of subjects referred to specialist cough clinics despite extensive investigations and it is likely that obstructive sleep apnoea is missed in some cases. It is important to recognise this condition early because of its implications for driving and operating machinery and associated cardiovascular morbidity if left untreated. CPAP is a very effective therapy for obstructive sleep apnoea associated cough, is well tolerated and resolution of cough was seen in all patients. Our preliminary series indicates that there is an association between cough and obstructive sleep apnoea. Placebo controlled trials with CPAP will need to be performed to establish the value of this treatment modality for obstructive sleep apnoea related cough.

## Competing interests

The author(s) declare that they have no competing interests.

## Authors' contributions

SSB conceived of the study, and participated in its design and coordination and wrote the manuscript. AJI, KC and GC participated in subject recruitment and clinical characterisation, and application of cough monitoring techniques to this study. SM participated in the cough monitoring of patients. MDLM and IDP participated in subject recruitment, clinical characterisation, analysis and writing up of the manuscript. All authors read and approved the final manuscript.

## Funding

none
